# Human Monoclonal Antibody Combination against SARS Coronavirus: Synergy and Coverage of Escape Mutants

**DOI:** 10.1371/journal.pmed.0030237

**Published:** 2006-07-04

**Authors:** Jan ter Meulen, Edward N van den Brink, Leo L. M Poon, Wilfred E Marissen, Cynthia S. W Leung, Freek Cox, Chung Y Cheung, Arjen Q Bakker, Johannes A Bogaards, Els van Deventer, Wolfgang Preiser, Hans Wilhelm Doerr, Vincent T Chow, John de Kruif, Joseph S. M Peiris, Jaap Goudsmit

**Affiliations:** **1**Crucell Holland B.V., Leiden, Netherlands; **2**Department of Microbiology, The University of Hong Kong, Queen Mary Hospital, Hong Kong Special Administrative Region of the People's Republic of China; **3**Institute for Medical Virology, Johann Wolfgang Goethe University, Frankfurt am Main, Germany; **4**Department of Microbiology, Yong Loo Lin School Faculty of Medicine, National University of Singapore, Singapore; Scripps Research InstituteUnited States of America

## Abstract

**Background:**

Experimental animal data show that protection against severe acute respiratory syndrome coronavirus (SARS-CoV) infection with human monoclonal antibodies (mAbs) is feasible. For an effective immune prophylaxis in humans, broad coverage of different strains of SARS-CoV and control of potential neutralization escape variants will be required. Combinations of virus-neutralizing, noncompeting mAbs may have these properties.

**Methods and Findings:**

Human mAb CR3014 has been shown to completely prevent lung pathology and abolish pharyngeal shedding of SARS-CoV in infected ferrets. We generated in vitro SARS-CoV variants escaping neutralization by CR3014, which all had a single P462L mutation in the glycoprotein spike (S) of the escape virus. In vitro experiments confirmed that binding of CR3014 to a recombinant S fragment (amino acid residues 318–510) harboring this mutation was abolished. We therefore screened an antibody-phage library derived from blood of a convalescent SARS patient for antibodies complementary to CR3014. A novel mAb, CR3022, was identified that neutralized CR3014 escape viruses, did not compete with CR3014 for binding to recombinant S1 fragments, and bound to S1 fragments derived from the civet cat SARS-CoV-like strain SZ3. No escape variants could be generated with CR3022. The mixture of both mAbs showed neutralization of SARS-CoV in a synergistic fashion by recognizing different epitopes on the receptor-binding domain. Dose reduction indices of 4.5 and 20.5 were observed for CR3014 and CR3022, respectively, at 100% neutralization. Because enhancement of SARS-CoV infection by subneutralizing antibody concentrations is of concern, we show here that anti-SARS-CoV antibodies do not convert the abortive infection of primary human macrophages by SARS-CoV into a productive one.

**Conclusions:**

The combination of two noncompeting human mAbs CR3014 and CR3022 potentially controls immune escape and extends the breadth of protection. At the same time, synergy between CR3014 and CR3022 may allow for a lower total antibody dose to be administered for passive immune prophylaxis of SARS-CoV infection.

## Editors' Summary

**Background.:**

Late in 2002, severe acute respiratory syndrome (SARS) emerged in the Guangdong province of China. In February 2003, an infected doctor from the province carried this new viral threat to human health to Hong Kong. Here, people staying in the same hotel caught the disease and took it to other countries. SARS was on the move, hitching lifts with international travellers. Because the virus responsible for SARS—SARS-CoV—spread by close person-to-person contact and killed 10% of the people it infected, health experts feared a world-wide epidemic. This was avoided by the World Health Organization issuing a global alert and warning against unnecessary travel to affected areas and by public-health officials isolating patients and their close contacts. By July 2003, the first SARS epidemic was over. 8,098 people had been infected; 774 people had died. Since then, sporadic cases of SARS have been contained locally.

**Why Was This Study Done?:**

The first epidemic of SARS was caused by an animal virus that became adapted to spread between people. There is no reason this process won't be repeated. If it is, stringent quarantine measures could again prevent a global epidemic, but at considerable economic cost. What is needed is a way to prevent SARS developing in healthy people who have been exposed to SARS-CoV and to treat sick people so that they are less infectious and can fight the virus. In this study, researchers have been investigating “passive immunization” as a way to limit SARS epidemics. In passive immunization, short-term protection against illness is achieved by injecting antibodies—proteins that recognize specific molecules (called antigens) on foreign organisms such as bacteria and viruses and prevent those organisms from causing disease. Antibodies for passive immunization can be isolated from blood taken from people who have had SARS, or they can be manufactured as so-called “human monoclonal antibodies” in a laboratory. One of these human monoclonal antibodies—CR3014—had been previously made and shown to prevent lung damage in ferrets infected with SARS-CoV and to stop the infected animals from infecting others. But for effective disease prevention in people, a single monoclonal antibody might not be enough. There are strains of SARS-CoV that CR3014 does not recognize and therefore cannot act against. Also, the virus can alter the antigen recognized by CR3014 when it is grown at a low antibody concentration, producing so-called escape variants; if this happens CR3014 can no longer prevent these escape variants from killing human cells.

**What Did the Researchers Do and Find?:**

The researchers tested how well a combination of two monoclonal antibodies controlled SARS-CoV killing of human cells. First, they showed that CR3014 escape variants all had the same small change in a part of the virus surface that interacts with human cells. CR3014 blocked this interaction in the parent SARS-CoV strain but not in the escape variants. They then made a new monoclonal antibody—CR3022—that prevented both the parent SARS-CoV stain and the CR3014 escape viruses from killing human cells. The two antibodies bound to neighboring parts of the virus surface, and both of them could bind at the same time. CR3022 also bound to surfaces of SARS-CoV strains to which CR3014 does not bind. And when they tried, the researchers could not generate any viral escape variants to which CR3022 was unable to bind. Finally, the effect of the two antibodies together on inhibition of SARS-CoV killing of human cells was more than the sum of their individual effects.

**What Do These Findings Mean?:**

A combination of two (or more) human monoclonal antibodies that recognize different parts of the SARS-CoV surface that interacts with human cells might be a good way to immunize people passively against SARS-CoV. It might minimize the possibility of escape variants arising, broaden the range of virus strains against which protection is provided, and reduce the amount of antibody needed for effective protection. Before the approach is tried in people, it will have to be tested in animals—results from experiments done on human cells in dishes are not always replicated in whole animals or people. If the approach passes further tests, the hope is that passive immunization of people with SARS and their close contacts might reduce disease severity in infected people and reduce viral spread as effectively as dramatic quarantine measures

**Additional Information.:**

Please access these websites via the online version of this summary at http://dx.doi.org/10.1371/journal.pmed.0030237.

• Medline Plus pages on SARS

• US Centers for Disease Control and Prevention information on SARS

• US National Institute of Allergy and Infectious Diseases factsheet about research on SARS

• Wikipedia page on SARS and monoclonal antibodies (note: Wikipedia is a free online encyclopedia that anyone can edit)

## Abstract 3

Two human monoclonal antibodies that bind to different parts of the viral glycoprotein spike show synergistic effects in virus neutralization and suppress the emergence of resistant virus in vitro.

## Introduction

Severe acute respiratory syndrome (SARS) is caused by a novel coronavirus (SARS-CoV) that spread in 2003 and 2004 from the Guangdong province of southern China to 32 countries in Asia, Europe, and North America, resulting in over 8,000 cases and 774 deaths [ 1, 2]. The main epidemic was stopped in late 2003 through internationally coordinated public health measures including early isolation of SARS patients and quarantine of contacts, at considerable economic costs to the affected countries [ 3– 5]. In 2003 and 2004 a number of isolated SARS cases occurred again in China and other Asian countries; some of these cases were linked to laboratory or unknown exposure, but others were community acquired and associated with exposure to the live game animal restaurant trade in Guangdong province [ 6, 7]. SARS-CoV-like viruses almost identical to a patient's isolate were found in palm civet cats investigated during the same period [ 7]. Phylogenetic analysis of approximately 100 SARS-CoV isolates from humans and civet cats collected from 2002 to 2004 revealed that SARS is a zoonotic disease that is evolving in palm civet and human hosts [ 7– 9]. Furthermore, presumably asymptomatic infection with SARS-CoV-like coronaviruses has repeatedly occurred in wild animal handlers in Guangdong province several years before the SARS epidemic [ 9]. Recently, bats have been identified as an important natural reservoir of the virus [ 10]. It is therefore unlikely that the mass culling of civets cats performed in southern China is sufficient to prevent further spillover of SARS-CoV or SARS-CoV-like viruses to the human population.

In the search for a vaccine against SARS, neutralizing antibodies have been raised successfully in experimental animals through immunization with whole killed virus or recombinant vaccine constructs expressing the SARS-CoV glycoprotein spike (S) [ 11– 13]. Correspondingly, studies of the immune responses in SARS patients have shown that the appearance of neutralizing antibodies coincides with a decrease of virus titers in serum, urine, stool, and nasopharyngeal secretions; in addition the beneficial effect of convalescent plasma in SARS patients has been reported [ 14– 16]. Neutralizing antibodies against SARS-CoV generated through experimental vaccination or monoclonal antibody (mAb) technology have repeatedly been shown in passive transfer experiments to protect mice from infection by reduction of viral replication [ 11, 17– 20]. In addition, the human mAb CR3014, generated by phage display, completely prevented lung pathology in the ferret model of SARS-CoV infection and abolished shedding of the virus in pharyngeal secretions [ 21]. CR3014 was shown to block interaction of the S1 subunit of the S protein with its cellular receptor ACE2 [ 22].

Because passive immunization using polyclonal sera has been reported to curb outbreaks of hepatitis A virus and to prevent infection with varicella-zoster virus, mAb prophylaxis may be an effective means of controlling a SARS outbreak [ 23, 24]. To this end, it is important that a mAb product offer sufficient breadth of protection against all relevant strains of SARS-CoV and prevent the selection of neutralization escape variants in the patient. In this study we therefore address both of these issues and present the characteristics of a combination of CR3014 with a new SARS-CoV neutralizing human mAb that was isolated from a convalescent patient. Because antibody-dependent enhancement of viral replication has been observed in macrophages for one member of the coronavirus family (feline infectious peritonitis virus), we also investigated whether a similar phenomenon occurs with SARS-CoV [ 25]. Infection of primary human macrophages with SARS-CoV has been reported to result in abortive infection with no infectious virus progeny produced [ 26]. We therefore studied whether virus infection in the presence of serial dilutions of CR3014 or serum from a convalescent SARS patient leads to productive virus infection.

## Methods

### Human Monoclonal Antibodies

The mAb CR3014 was isolated from a semisynthetic single-chain variable antibody fragment (scFv) phage display library, expressed as human IgG1 molecules and purified as described previously [ 22, 27]. An immune scFv phage display library was constructed from lymphocytes of a convalescent SARS patient from Singapore essentially as described [ 28]. From this library, CR3022 scFv was selected for binding to UV-inactivated SARS-CoV, essentially as described [ 22]. SARS-CoV (Frankfurt 1 strain [FM1]) was prepared as described and UV-irradiated for 15 min (UVB radiation, 280–350 nm; λ _max_, 306 nm) at 4 °C. CR3022 scFv was converted into a human IgG1 format and expressed and purified as described. Anti-rabies mAb CRJA served as negative control.

### Virus Neutralization Assay and Determination of Antibody Potency

Research involving SARS-CoV virus was performed under biosafety level 3 containment. SARS-CoV strain HKU-39849 at 2 × 10 ^3^ TCID _50_/ml in 100 μl was mixed with an equal volume of serially diluted mAb in maintenance medium (MM, MEM supplemented with 1% v/v FCS) and incubated for 1 h at 37 °C. Subsequently, the mixture was inoculated in octuplicate onto 96-well plate containing a 80% confluent culture of fetal rhesus kidney cells (FRhK-4; American Type Culture Collection, Manassas, Virginia, United States; #CRL-1688). The FRhK-4 cells were cultured at 37 °C and observed after 3–4 d for the development of CPE. The mAb concentrations required for 50% and 100% neutralization were calculated from two-fold dilution series according to the Spearman-Karber formula. To quantitatively compare the neutralizing potency of a CR3014/CR3022 combination with the individual potencies of the mAbs, they were mixed in an equimolar ratio and the 100% and 50% neutralization potency of a serial dilution of the mix was determined. The concepts of the combination index (CI) and dose reduction index (DRI) were used to quantify synergistic effects as described previously [ 29, 30].

### Generation and Characterization of Neutralization Escape Variants

Serial dilutions ranging from 10 ^−1^ to 10 ^−8^ of SARS-CoV strain HKU-39849 were incubated in the presence of a constant amount of CR3014 (20 μg/ml) or CR3022 (60 μg/ml), for 1 h at 37 °C and 5% CO _2_ before addition to wells containing FRhK-4 cells. The virus-mAb mixture was incubated with cells for 1 h at 37 °C and 5% CO _2_, then the virus was removed and the cells were washed twice with medium. Finally, cells were incubated for 2 d in the presence of the above concentrations of mAb in 0.5 ml of medium. Supernatant of cells incubated with highest virus dilution showing CPE, containing the potential escape viruses, was harvested and stored at 4 °C until further use. Virus samples were freeze-thawed once, serial dilutions were prepared in medium and added to wells containing FRhK-4 cells, and the dilutions incubated for 1 h at 37 °C and 5% CO _2_ in the presence of mAb. Wells were then overlaid with agarose containing mAbs at the above concentration and incubated for 3–5 d at 37 °C and 5% CO _2_. Individual escape virus plaques were picked using a Pasteur pipette and freeze-thawed once, and the escape viruses were amplified on FRhK-4 cells. To identify possible mutations in the SARS-CoV spike protein of each of the escape viruses, the nucleotide sequence of the SARS-CoV S open reading frame (ORF) was determined. Viral RNA of each of the escape viruses and wild-type SARS-CoV virus was isolated and converted into cDNA by standard RT-PCR. Subsequently, the cDNA was used for nucleotide sequencing of the SARS-CoV S ORF in order to identify mutations. Secondly, the neutralization index was determined for each of the escape viruses. Therefore, each escape virus and wild-type SARS-CoV (100 TCID _50_) was incubated for 1 h at 37 °C and 5% CO _2_ with mAb at the above concentration before addition to FRhK-4 cells. The virus was allowed to attach to the cells for 1 h at 37 °C and 5% CO _2_, after which the inoculum was removed and cells were washed twice with medium before being replenished with medium containing mAb. After a 2 d incubation at 37 °C and 5% CO _2_ the medium was harvested and the TCID _50_/ml of each virus was determined. The neutralization index was determined by subtracting the number of infectious virus particles (expressed as log TCID _50_/ml) produced in FRhK-4 cell cultures infected with virus in the presence of mAb from the number of infectious virus particles (log TCID _50_/ml) produced in FRhK-4 cell cultures infected with virus alone. An index lower than 2.5 was considered evidence of escape.

### Recombinant SARS-CoV Glycoprotein Spike Fragments

The generation of the plasmids encoding recombinant spike (S) fragments comprising residues 318–510 of the human SARS-CoV strains FM1, GZ02, Sin3408, Shanghai LY, GZ-C, Sino1–11, BJ302 clone 2, GD03T0013, GD01, and the palm civet cat derived strain SZ3 (AY304486), as well as expression and purification of the recombinant proteins have previously been described [ 22].

### Epitope Mapping using ELISA

Direct ELISA using the recombinantly expressed S fragment comprising amino acids 318–510 (S318–510) was performed with the mAbs as described [ 22]. Competition ELISA was performed as follows. Anti-Myc-captured S318–510 fragment of the Frankfurt 1 strain was incubated with nonsaturating amounts of biotinylated IgG in the presence or absence of competing IgG. Bound biotinylated IgG was detected with streptavidin-conjugated-HRP (BD Pharmingen, San Diego, California, United States). Alternatively, S318–510 was captured by CR3014 or CR3022, detected using a biotinylated CR3014 and CR3022, and developed as described above.

### BIAcore Analysis

Surface plasmon resonance analyses were performed at 25 °C on a BIAcore3000 (Biacore AB, Uppsala, Sweden). CM5 sensorchips and running buffer HBS-EP were from Biacore AB. Recombinant S318–510 was immobilized to CM5 chips using an amine coupling procedure resulting in a response level of approximately 1,000 resonance units. Kinetic analysis was performed to determine the association rate constant ( *k*_a_), dissociation rate constant ( *k*_d_), and the affinity ( *K*_D_) of the mAbs. Therefore, a concentration series of 0.4–250 nM IgG was prepared using two-fold dilutions in HBS-EP. Samples were injected in duplicate at a flow rate of 30 μl/min (injection time, 2 min; dissociation time, 5 min). The sensor chip surface was regenerated with a 5 μl pulse of 5 mM NaOH. Biacore evaluation software (BIAevaluation, version 3.2) was used to fit the association and dissociation curves of all concentrations injected.

### Immune Enhancement Assay of SARS-CoV Replication in Primary Human Macrophages

Preparation of primary human monocytes/macrophages and their infection with SARS-CoV was performed as previously described [ 26]. To investigate the effect of subneutralizing doses of anti-SARS Abs on viral infection in macrophages, 300 μl of serially diluted mAb in MM medium, convalescent serum from a SARS-CoV exposed individual, or serum from a healthy individual was mixed with 300 μl of SARS-CoV. MM medium mixed with SARS-CoV served as the virus control. The virus/mAb mixtures and virus/serum mixtures were incubated for 1 h at 37 °C. Then, 250 μl of the mixtures was added to duplicate wells containing macrophages. After 1 h of virus adsorption at 37 °C, the virus inoculum was removed, infected cells were washed with macrophage SFM culture medium and incubated in macrophage SFM medium supplemented with 0.6 μg/ml penicillin and 60 μg/ml streptomycin. Samples of the culture supernatants were collected at days 0, 1, 2, 3, 5, and 7 postinfection and stored at −70 °C for virus titration experiments. SARS-CoV was titrated and the TCID _50_ determined essentially as described. RNA was isolated from infected macrophages at 6 and 24 hours postinfection using the RNeasy Mini kit (Qiagen, Valencia, California, United States) according to the manufacturer's instructions. Quantification of positive- and negative-strand viral RNA was performed by real-time quantitative RT-PCR targeting the *ORF1b* gene, as described previously [ 31]. The SARS-CoV RNA levels were normalized for the levels of *β-actin* mRNA.

## Results

### All CR3014 Neutralization Escape Variants of SARS-CoV Display a P462L Mutation in the Spike Glycoprotein

**Figure 1:**
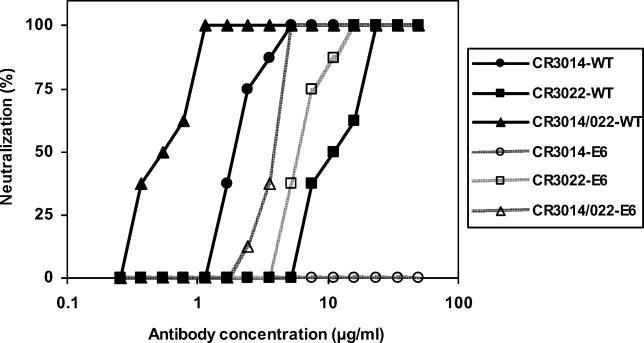
Neutralization of Wild-Type SARS-CoV and a CR3014-Neutralization Escape Variant (E6) with mAbs CR3014 and CR3022 Individually and in Combination Neutralization of 100 TCID _50_ of each virus was performed in octuplicate. Data are also shown in Table 1.

**Figure 2:**
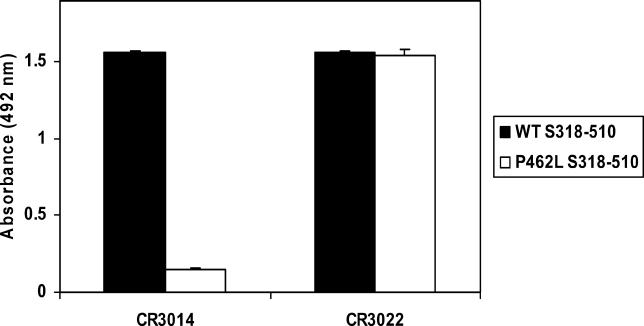
Binding of mAbs CR3014 and CR3022 to Recombinant Wild-Type and P462L-Substituted S318–510 Fragments Analyzed by ELISA Bars represent the means ± standard deviation.

Full neutralization curves of wild-type SARS-CoV and one CR3014-neutralization escape virus with CR3014 and CR3022 are shown in Figure 1. All five neutralization escape variants were completely refractory to neutralization with CR3014. Sequencing of the complete spike gene of these variants isolated from two separate viral cultures ( *n* = 5) revealed that all carried a single point mutation at amino acid position 462 (proline to leucine, P462L), which abolished binding of CR3014 to the recombinantly expressed receptor-binding domain (RBD) fragment (S318–510) displaying this mutation ( Figure 2). This mutation has to date been observed neither in SARS-CoV isolates from human patients nor in SARS-CoV-like coronaviruses found in civet cats. In two independent experiments, repeated passage of SARS-CoV in the presence of up to 60 μg/ml CR3022 did not result in the generation of neutralization escape variants (unpublished data).

**Table 1:**
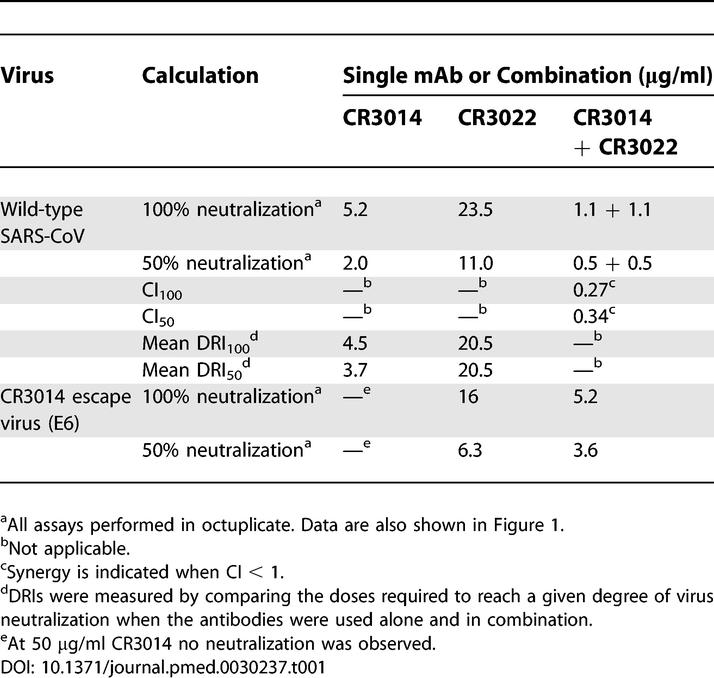
Synergy of CR3014 and CR3022 for SARS-CoV Neutralization and Coverage of CR3014 Escape Viruses by CR3022

### Human mAb CR3022 Neutralizes CR3014 Escape Variants of SARS-CoV by Binding Noncompetitively to the RBD of the Spike Glycoprotein

**Figure 3:**
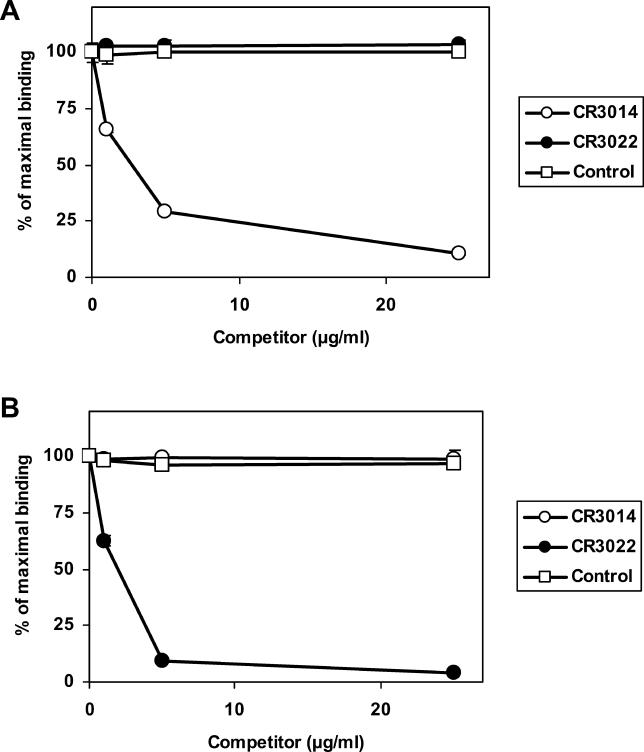
Competition ELISA on Immobilized S318–510 Binding of biotinylated CR3014 (A) and CR3022 (B) was analyzed in the presence of competitor CR3014 (open circles), CR3022 (closed circles), and control mAb (open squares). Binding is expressed as percentage of binding without competitor.

An antibody phage display library was constructed from RNA isolated from the lymphocytes of a convalescent SARS patient originating from Singapore. Antibody CR3022 was isolated which completely neutralized the SARS-CoV strain HKU-39849 and the CR3014 escape variants at a concentration of 23.5 μg/ml ( Table 1). CR022 was shown in ELISA to bind noncompetitively with CR3014 to the recombinantly expressed RBD of the S glycoprotein of wild-type SARS-CoV (strain Frankfurt 1) and also to the mutated RBD (P462L) of the escape variants ( Figures 2 and 3), which were completely neutralized by 16 μg/ml of this mAb. In contrast to CR3014, CR3022 did not prevent binding of a recombinant S fragment composed of amino acids 1–565 to Vero cells (unpublished data). The sequences of the variable regions of the heavy and light chains of CR3022 were deposited at GenBank.

### CR3022 Shows Extended Reactivity with Recombinantly Expressed S Fragments of Different SARS-CoV Strains

**Figure 4:**
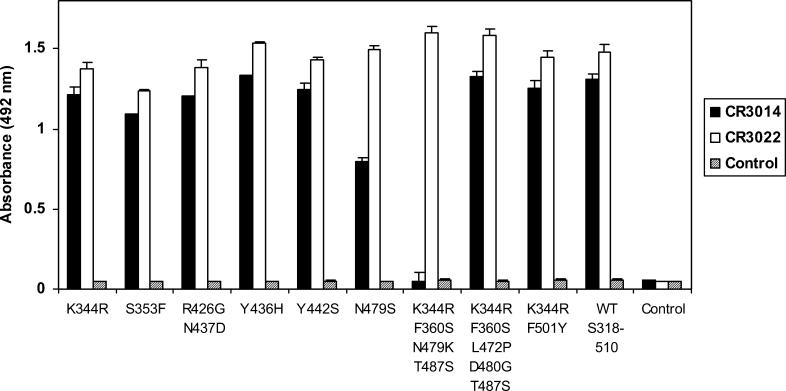
Binding of Human mAbs to S318–510 Fragments in ELISA Wild-type and variant fragments were synthesized according to published sequences of SARS-CoV strains. Wild-type S318–510 from strain Frankfurt 1, variant fragments from strains GZ02 (K344R), Sin3408 (S353F), Shanghai LY (R426G and N437D), GZ-C (Y436H), Sino1–11 (Y442S), BJ302 cl. 2 (N479S), SZ3 (K344R, F360S, N479K, and T487S), GD03T0013 (K344R, F360S, L472P, D480G, and T487S), and GD01 (K344R and F501Y). Bars represent the means ± standard deviation.

In addition to the fragments recognized by CR3014, CR3022 also binds to the RBDs of SARS-CoV-like isolate SZ3 isolated from a civet cat and from a human SARS-CoV with a mutation of asparagine to serine at amino acid 479 (N479S), which were not or only partially recognized by CR3014, respectively ( Figure 4).

### CR3014 and CR3022 Neutralize SARS-CoV Synergistically

To evaluate whether CR3014 and CR3022 would neutralize SARS-CoV in an additive or synergistic manner, we used the classic method of titrating the neutralizing potency of an equimolar mixture of the antibodies and comparing the dose response with that from neutralization assays performed with the individual antibodies ( Figure 1). The data were analyzed by applying the median effect principle as formulated by Chou and Talalay [ 29]. A combination index (CI) of less than 1, indicating synergistic neutralization, was observed in three independent experiments for both 50% and 100% neutralization of wild-type SARS-CoV ( Table 1). As is evident from the DRIs, the mixture of antibodies reduced the required antibody dose especially for CR3022 considerably (DRI = 20.4). Interestingly, CR3014 also slightly enhanced neutralization of the escape viruses by CR3022 ( Figure 1).

### Simultaneous Binding of mAbs CR3014 and CR3022 to Recombinant S318–510 Fragment Does Not Change Their Affinities

To investigate changes of affinities as a possible mechanism of synergy, the *K*_D_ for CR3014 and CR3022 binding sequentially or simultaneously to recombinant RBD was investigated. The individual *K*_D_ values for CR3014 and CR3022 were 16.3 nM and 0.125 nM, respectively; for the antibodies binding simultaneously was 5.71 nM; and for binding of CR3014 to CR3022-saturated S318–510 was 14.8 nM. Compared to the dose reduction indices of 4.5 and 20.5 for CR3014 and CR3022, respectively, neither simultaneous nor sequential binding of the mAbs resulted in changes of *K*_D_ that could explain their synergistic neutralizing action through cooperative binding.

### SARS-CoV Does Not Replicate in Human Macrophages in the Presence of mAb CR3014 or Convalescent Serum

**Figure 5:**
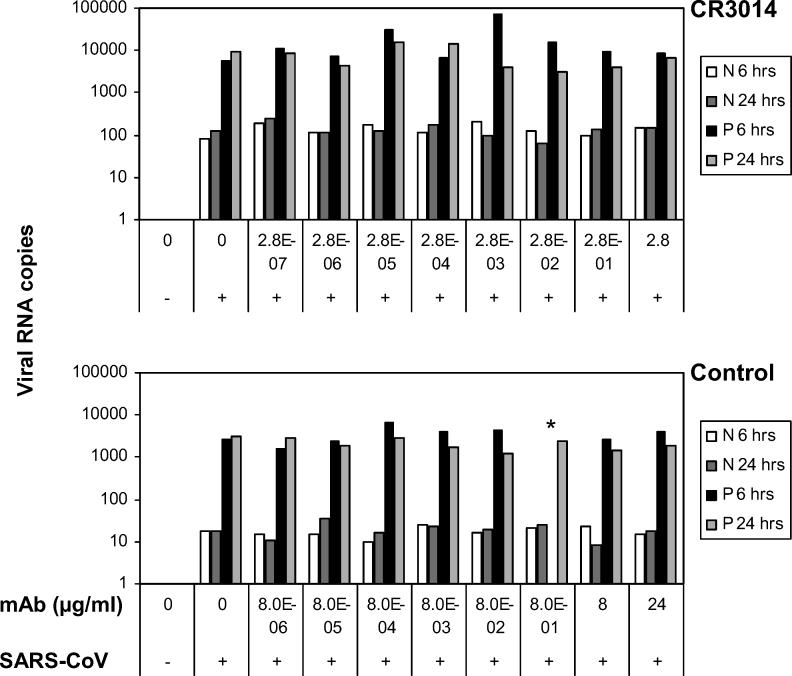
Infection of Differentiated Human Macrophages with SARS-CoV (HKU-39849) in the Presence of CR3014 or a Control mAb One representative experiment of three independent experiments is shown. Total RNA was extracted at 6 and 24 hours postinfection, and the copy number of positive (P) and negative (N) strand RNA of SARS-CoV *ORF1b* was determined by real-time quantitative RT-PCR and normalized for the levels of *β-actin* mRNA. The bars represent mean of duplicate viral load titrations. In the control experiment, * indicates an aberrant result.

SARS-CoV did not replicate to measurable titers after day 2 on macrophages in the absence or presence of serial dilutions of mAb CR3014, a control mAb, or serum from a convalescent patient (unpublished data). Figure 5 shows the detection of SARS-CoV positive (P) or negative (N) strand RNA (replication intermediate) by RT-PCR in the same assay. Positive-strand RNA is detectable at 6 and 24 h postinfection, indicating uptake of virus by the macrophage. In the presence of 2.8 × 10 ^−5^ to 2.8 × 10 ^−3^ μg/ml of CR3014 a slight increase in virus uptake into macrophages is suggested by the increased copy number of positive-strand RNA ( Figure 5). In contrast, negative-strand RNA, which indicates viral replication, is detectable at much lower levels that are constant over time, with no difference between the virus incubated with CR3014 or control antibody. Taken together, these results show that with or without monoclonal or polyclonal antibody, macrophages take up SARS-CoV, but this uptake does not lead to the productive virus replication and release of infectious virus.

## Discussion

Our results show that SARS-CoV can be neutralized synergistically by human mAbs targeting the receptor-binding domain of the virus. The combination of two mAbs expanded the breadth of protection and controlled potential immune escape variants while reducing the total antibody concentration required to neutralize the virus. In view of the proven efficacy of passive prophylaxis of SARS-CoV infection in animal models using single mAbs, our data provide a rationale to develop cocktails of mAbs for human SARS prophylaxis.

Although the SARS epidemic of 2003 was successfully contained, the risk of its re-emergence remains from its natural animal reservoir, from intermediate hosts such as civet cats in live animal markets, or from laboratory associated infections. The re-emergence and community spread of SARS in Beijing in February 2004 required the quarantine of large numbers of contacts; the social and economic consequences of such measures are considerable. The availability of prophylactic and therapeutic measures against SARS-CoV would greatly assist the control of any future re-emergence of the virus, and their development therefore remains a high priority [ 32].

With respect to vaccine development, considerable progress has been made in understanding the immune response against the virus and defining correlates of protection. Patients with high viral loads and shedding of SARS-CoV in pharyngeal secretions have elevated mortality [ 33]. Correspondingly, the development of neutralizing antibody titers correlates with a decrease of virus titers in serum, urine, stool, and nasopharyngeal secretions [ 15]. Progress has also been made in understanding the target of the neutralizing immune response through immunization with whole killed SARS-CoV and recombinant vectors expressing the S glycoprotein and other structural proteins [ 11– 13]. Neutralizing antibodies seem to be predominantly directed against a domain of the S1 subunit of the spike protein, defined by amino acids 318–510, which is responsible for interaction of the virus with its cellular receptor ACE2 [ 34– 37]. Mapping of this RBD using a panel of mouse mAbs has led to the identification of six distinct neutralizing epitopes, which are all conformation-dependent [ 38]. The neutralizing potency of the mAb correlated directly with their ability to block the RBD-ACE2 interaction in recombinant assays; however, several neutralizing mAbs were also identified that bind to two distinct epitopes of the RBD without inhibiting the interaction with ACE2.

The concept of using polyclonal or monoclonal antibodies with neutralizing activity against SARS-CoV for passive immunization to control both development of severe disease and transmission from human to human of the virus is based on several observations. The use of convalescent serum in SARS patients was reported to be associated with possible benefit and in any event was not associated with adverse effects [ 14, 16] *.* In consequence, commercial production of SARS antiserum from human donors has been initiated in China [ 39]. Passive transfer of immune serum generated through immunization of animals with whole killed virus or vector-expressed full-length S or S fragments has reduced viral replication in a mouse model of SARS-CoV infection [ 11, 18]. Human mAbs have been generated through phage display, immortalization of human B-cells from SARS-patients, or immunization of transgenic mice, and were shown to display protective efficacy in the mouse model [ 17, 19, 20]. In addition, the human mAb CR3014 reduced SARS-CoV replication in the lungs of infected ferrets, completely prevented the development of lung lesions, and abolished viral shedding in the pharyngeal secretions of the animals [ 21]. Because SARS-CoV is spread by the respiratory and the fecal-oral route, the observation that passive immunization is successful in controlling outbreaks of hepatitis A virus and protecting against varicella-zoster and respiratory syncytial virus infection argues for the feasibility of an emergency immune prophylaxis during a SARS outbreak [ 23, 24, 40].

Monoclonal antibody prophylaxis against SARS must meet certain criteria to be effective. The breadth of protection of a single mAb may not be sufficient to protect against all clinically relevant strains of the virus, and it has been suggested that genotyping of SARS-CoV in case of a new outbreak may be required to select for optimally neutralizing mAbs [ 19]. A comprehensive analysis of the variability of approximately 100 full-length genomes of SARS-CoV isolates obtained from human patients and civet cats between 2002 and 2004 revealed that the S glycoprotein, especially the RBD, is under strong positive selective pressure during the transition from animal-to-animal to human-to-human transmission, because of differences in the ACE2 receptors of civet cats and humans [ 7]. The highest variability of the RBD is observed within the group of SARS-CoV-like viruses isolated from animals, with no data as yet available concerning the receptor specificity of the newly discovered bat-CoV [ 10]. Interestingly, patients infected with SARS-CoV-like viruses had a milder course of disease and did not transmit the viruses to other persons, presumably because of insufficient adaptation of the civet cat virus RBD to human ACE2, which is required for productive infection of human cell lines [ 19, 41, 42]. The RBD of SARS-CoV isolates from the next SARS epidemic will therefore likely be very similar to the isolates from 2003. The RBD of human SARS-CoV isolates is highly conserved and alignment of 114 sequences published in GenBank revealed only eight different S sequences not identical to the FM1 strain [ 22]. Of all human isolates SARS-CoV-like isolate GD03T0013, which was obtained from a patient in 2003, shows the highest RBD divergence and closest relatedness to civet cat strains [ 7]. Human mAb CR3014 binds well to recombinantly expressed RBDs representing seven of the RBD variants, including GD03T0013, but less to a variant with a N479S mutation found in human isolate BJ302 cl.2, and not at all to an RBD derived from the civet cat SARS-CoV-like isolate SZ3. Antibody CR3022, derived by phage display from a convalescent SARS patient, binds well to the latter two variants in addition to the others. A combination of CR3014 and CR3022 may therefore offer sufficient protection not only against SARS-CoV strains but also against SARS-CoV-like viruses originating from civet cats.

Viral replication in the presence of a mAb, especially at subneutralizing concentrations, carries the risk of selecting neutralization escape variants. We therefore analyzed escape variants of SARS-CoV strain HKU-39849 generated under selective pressure of CR3014 or CR3022, which was unsuccessful for the latter. All CR3014 escape variants had the same single amino acid exchange in the RBD (P462L), which has not been reported in any SARS-CoV or SARS-CoV-like virus to date. This variant was successfully neutralized with CR3022, extending the breadth of protection of this antibody combination from naturally occurring variants also to possible CR3014 immune escapes. A similar strategy was previously pursued to develop a mAb product for postexposure prophylaxis of rabies [ 43]. Because neutralization escape in vitro may not accurately reflect the in vivo situation, the CR3014/CR3022 combination needs to be tested at subneutralizing concentrations in animal models of SARS-CoV infection.

Further investigation of the CR3014/CR3022 combination revealed that the antibodies neutralize wild-type SARS-CoV synergistically in vitro, with DRIs of about four and 20 for the two mAbs, respectively. Synergism of neutralizing mAbs has not yet been reported for SARS-CoV, but was observed for combinations of two, three, or four mAbs directed against different epitopes on the HIV-1 envelope glycoprotein, leading to a two- to ten-fold increase of neutralization titers [ 30, 44, 45]. Mechanistically, cooperative binding of mAbs may induce conformational changes in the antigen thereby altering affinities (allosteric effect), lead to intermolecular interaction of bivalent antibodies, or result in interaction of the Fc regions of antibodies brought into close contact [ 46]. As determined by ELISA, CR3014 and CR3022 bind simultaneously to the RBD of the S1 subunit of the glycoprotein S of SARS-CoV. However, measuring separate, sequential, and simultaneous binding of the mAbs in Biacore did not reveal enhanced affinity of the antibody mixture over the individual affinities, especially for CR3022. Another possible explanation for synergism could be increased breadth of protection against different quasispecies of a viral isolate [ 30]. Heterogeneous sequences have been detected through direct sequencing of SARS-CoV RNA recovered from single patient samples, and adaptive mutations induced by repeated cell culture passage have been reported [ 47– 49]. It should therefore be formally investigated whether SARS-CoV quasispecies formation in cell culture may influence the outcome of virus neutralization assays. Lastly, CR3022 could interfere with binding of the S1 subunit to a known or unknown coreceptor of the virus [ 50]. The mechanism by which the two mAbs neutralize SARS-CoV synergistically therefore remains presently elusive.

It was previously shown that CR3014 protects ferrets from challenge with SARS-CoV at a dose of 10 mg/kg by completely preventing lung pathology and abolishing shedding of the virus [ 21]. This antibody dose compares well with the required dosage of Palivizumab (15 mg/kg), which protects at-risk infants from infection with respiratory syncytial virus and is currently the only licensed antiviral monoclonal antibody [ 40]. However, the required amount of CR3014 for protecting a standard 70 kg adult is relatively high, and it would be desirable to use a lower dose for economic reasons. Furthermore, the even lower potency of CR3022 precludes its use as a stand-alone prophylaxis. Therefore, the observed four- to twenty-fold dose reduction of the individual mAbs in the CR3014/CR3022 mixture may hold an attractive option to reduce the total antibody dose required for neutralizing the virus in vivo while at the same time expanding the breadth of protection.

In this study, we also addressed the potential problem of antibody-dependent enhancement (ADE), which is a well-recognized phenomenon observed in infections with another coronavirus, feline infectious peritonitis virus. Both immunization and passive transfer of antibody was shown to mediate this phenomenon and mAbs have been used to map neutralizing and enhancing epitopes on the spike glycoprotein of feline infectious peritonitis virus [ 25, 51]. There is no direct evidence of ADE linked to SARS-CoV infection in humans to date, and infection of human macrophages with SARS-CoV was previously shown to be abortive [ 25]. However, it was recently shown, in a viral pseudotype assay expressing the full-length spike glycoproteins of SARS-CoV-like viruses, that these cannot be neutralized with either homologous or heterologous mouse immune sera, and that both the sera and human mAbs neutralizing SARS-CoV enhance infectivity of the pseudotype for the human adenocarcinoma cells used as indicator system [ 34]. Given that ADE in feline infectious peritonitis virus infection is mediated by increased macrophage uptake of virus in the presence of neutralizing antibody, we performed human macrophage infectivity assays in the presence of CR3014 and human convalescent serum. In this type of assay, ADE of Dengue and Ross River virus infection has previously been demonstrated [ 52]. The addition of varying concentrations of CR3014 or convalescent SARS serum to SARS CoV did not convert the abortive infection into a productive one, reducing the likelihood that ADE will be observed in vivo after passive immunization.

In conclusion, we propose that a combination of neutralizing mAbs targeting the RBD of SARS-CoV has the potential to control SARS-CoV infection with a high level of efficacy and safety, and possibly at reduced economic cost. Isolation of suspected SARS cases and quarantine of exposed contacts have proved effective in controlling epidemics. However, the direct and indirect costs involved in quarantine measures are very substantial [ 4]. Monoclonal antibody prophylaxis (ring vaccination) in case of a SARS outbreak may prove more cost effective.

## Supporting Information

Alternative Language Abstract S1 Chinese Translation of the Abstract (21 KB DOC)

### Accession Numbers

The GenBank ( http://www.ncbi.nlm.nih.gov) accession number of SARS-CoV strain HKU-39849 is AY278491. The sequences of the CR3022 variable regions of the heavy and light chains are deposited in GenBank under accession numbers DQ168569 and DQ168570, respectively.

## Abbreviations

ADE: antibody-dependent enhancement
CI: combination index
DRI: dose reduction index
mAb: monoclonal antibody
ORF: open reading frame
RBD: receptor-binding domain
S: spike
SARS: severe acute respiratory syndrome
SARS-CoV: SARS coronavirus
scFv: single-chain variable antibody fragment
