# Supplementary material for: Human Monoclonal Antibody Combination against SARS Coronavirus: Synergy and Coverage of Escape Mutants
Source: PLoS Med. 2006 Jul 4;3(7):e237. doi: 10.1371/journal.pmed.0030237 (PMC1483912; doi:10.1371/journal.pmed.0030237)
Supplement: Supplementary file 1 [file pmed.0030237.sd001.doc]

背景﹕實驗性動物模型曾証明人類的單株中和抗體可控制SARS冠狀病毒的感染，但如要採用此種抗體作為預防方法，應同時採用多種單株抗體來控制不同的SARS冠狀病毒株，此亦可減低病毒表面蛋白變異的可能。

方法和研究結果﹕人類單株中和抗體CR3014在白鼬的身上有保護作用，我們採用這抗體在體外實驗中做出多個逃脫突變種。這些突變種的刺突蛋白全都帶有P462L的變異。體外實驗亦証明P462L的變異能避免抗原和抗體的結合。我們因此亦從一位已康復的SARS病人身上找出一種對CR3014有互保作用的單株抗體CR3022。此新單株抗體能中和CR3014 所產生的逃脫突變種。在競爭實驗中，CR3014 和CR3022 不會有競爭作用，CR3014 亦不能產生逃脫突變種。CR3014 和CR3022 亦有協同作用，而所用劑量也可因此而減少，巨噬細胞並沒有因此兩種單株而產生有感染性的SARS病毒。

結論﹕同時採用CR3014和CR3022單株抗體不但能擴大保護效能，亦能減低產生逃脫變異種的可能性，也可減低藥物服用的劑量。
